# Expert consensus of the Spanish Society of Pathology and the Spanish Society of Medical Oncology on the determination of biomarkers in pancreatic and biliary tract cancer

**DOI:** 10.1007/s12094-022-02873-0

**Published:** 2022-08-25

**Authors:** Ruth Vera, Carolina Ibarrola-de-Andrés, Jorge Adeva, Judith Pérez-Rojas, Pilar García-Alfonso, Yolanda Rodríguez-Gil, Teresa Macarulla, Teresa Serrano-Piñol, Rebeca Mondéjar, Beatriz Madrigal-Rubiales

**Affiliations:** 1 Navarra University Hospital, Spanish Society of Medical Oncology (SEOM), Calle Irunlarrea, 3, Navarra, 31008 Pamplona, Spain; 2 12 de Octubre University Hospital, Spanish Society of Pathology (SEAP), Madrid, Spain; 3 12 de Octubre University Hospital, Spanish Society of Medical Oncology (SEOM), Madrid, Spain; 4 La Fe University and Polytechnic Hospital, Spanish Society of Pathology (SEAP), Valencia, Spain; 5 Gregorio Marañón University Hospital, Spanish Society of Medical Oncology (SEOM), Madrid, Spain; 6 Vall d’Hebron University Hospital, Spanish Society of Medical Oncology (SEOM), Barcelona, Spain; 7 Bellvitge University Hospital, Spanish Society of Pathology (SEAP), Barcelona, Spain; 8 La Princesa University Hospital, Spanish Society of Medical Oncology (SEOM), Madrid, Spain; 9 Río Hortega University Hospital, Spanish Society of Pathology (SEAP), Valladolid, Spain

**Keywords:** Molecular diagnosis, Targeted therapies, Prognostic value, Predictive value, Pancreatic cancer, Biliary tract cancer

## Abstract

Pancreatic cancer and biliary tract cancer have a poor prognosis. In recent years, the development of new diagnostic techniques has enabled the identification of the main genetic alterations involved in the development of these tumours. Multiple studies have assessed the ability of certain biomarkers, such as *BRCA* in pancreatic cancer, *IDH1* or *FGFR2* in biliary tract cancer and microsatellite instability or *NTRK* fusions in an agnostic tumour fashion, to predict response to treatment.

In this consensus, a group of experts selected by the Spanish Society of Medical Oncology (SEOM) and the Spanish Society of Pathology (SEAP) reviewed the role played by these mutations in the process of carcinogenesis and their clinical implications. As a result, this article proposes a series of recommendations to optimize the determination of these biomarkers to help standardize the diagnosis and treatment of these tumours.

## Introduction

Pancreatic and biliary tract cancers (BTCs) have a poor prognosis and are leading causes of cancer-related death [1]. Pancreatic cancer was responsible for 6.7% of cancer deaths in Spain in 2020, and BTC accounted for 4.9% [2].

Advances in diagnostic techniques and molecular biology in recent years have enabled a better understanding of the main molecular alterations involved in the development of these tumours. This consensus reviews the main recommendations regarding the determination of these molecular alterations in pancreatic and BTCs, the frequency of these alterations and the role these alterations play in the process of carcinogenesis, as well as their clinical implications.

Multiple studies have explored predictive biomarkers of responses to specific therapies (chemotherapy, immunotherapy or targeted therapy). The most prominent of these biomarkers in pancreatic cancer are breast cancer gene (*BRCA*) 1 and 2 mutations, which are associated with greater therapeutic benefit under treatment with platinum-based chemotherapy and poly-ADP-ribose polymerase (PARP) inhibitors [3, 4]. For BTC, mutations in isocitrate dehydrogenase-1 (*IDH1*) have been associated with greater clinical benefits with ivosidenib [5]. In turn, fusions or rearrangements of fibroblast growth factor receptor-2 (*FGFR2*) have been associated with greater sensitivity to treatment with selective *FGFR* inhibitors [6].

Some of these tumour biomarkers have been studied agnostically, such as microsatellite instability-high (MSI-H) or neurotrophic tyrosine receptor kinase (*NTRK*) fusions as predictive factors of the response to immunotherapy and to tropomyosin kinase receptor inhibitors, respectively [7, 8].

This consensus document of the Spanish Society of Medical Oncology (SEOM) and the Spanish Society of Pathology (SEAP) proposes, based on current scientific evidence, several recommendations for these molecular biomarkers to standardize diagnostic processes involving biological samples in health centres.

## Clinical aspects

### Pancreatic cancer

Advanced pancreatic cancer has two standard first-line treatments: the combination of gemcitabine and nab-paclitaxel and the combination of folinic acid, 5-fluorouracil, irinotecan and oxaliplatin (FOLFIRINOX) [9, 10]. Clinical aspects guide the choice of one or the other. The FOLFIRINOX regimen is preferred in young patients in good general condition.

Retrospective data have shown that patients with pancreatic cancer carrying mutations in DNA repair genes have better survival if treated with platinum-based chemotherapy [4]. Thus, although the data are not prospectively validated, the presence of mutations in DNA repair genes is a predictive biomarker for the response to platinum-based chemotherapy.

The POLO study, a phase III clinical trial, evaluated the efficacy of olaparib as a maintenance treatment in patients with metastatic pancreatic cancer who carry a germinal mutation in *BRCA1* or *BRCA2* [3]. This study included 3,315 patients, of whom 7.5% carried such a mutation. Patients were treated with platinum-based chemotherapy for a minimum of 16 weeks, and more than 80% received FOLFIRINOX. A total of 154 patients who did not progress after chemotherapy were randomized to be treated with olaparib (300 mg/12 h) or placebo. The outcomes of this study were positive, achieving its main objective, i.e. an increase in progression-free survival, with a median of 7.4 months in the group treated with olaparib and 3.8 months in the placebo group (hazard ratio [HR]: 0.53). The study also reported a higher response rate and longer response duration for the group treated with olaparib than for the placebo group (23% and 24.9 months versus 12% and 3.7 months, respectively). However, no differences were observed in terms of overall survival (OS) between the two treatment groups (18.9 months compared to 18.1 months; HR: 0.91). Therefore, the presence of a germinal mutation in *BRCA1* or *BRCA2* is a predictive biomarker of response to olaparib if platinum-based chemotherapy has been administered without progression. On the basis of these results, the American Food and Drug Administration (FDA) and the European Medicines Agency (EMA) have approved the use of olaparib as a maintenance treatment in these patients.

A total of 93-95% of patients with pancreatic cancer have a mutation in the kirsten rat sarcoma virus (*KRAS*) gene*,* one of the leading disease genes [11, 12]. Some publications indicate that young populations (< 50 years) with pancreatic cancer more frequently present the nonmutated *KRAS* gene [12].

Different publications indicate a greater proportion of molecular alterations that are potential therapeutic targets in patients without *KRAS* mutations (native) than in populations with pancreatic cancer and *KRAS* mutations. Examples of these alterations are pathogenic variants at the germinal level in different genes, such as *BRCA1, BRCA2* or partner and localizer of *BRCA2* (*PALB2*)*,* or somatic-level fusions in neuregulin 1 (*NRG1*), rearranged during transfection (*RET*) or *NTRK*. Some clinical studies have shown promising antitumor activity with drugs that act directly or indirectly on the oncogenic molecular pathways associated with these fusions [7, 13, 14].

Immunotherapy, thus far, has not demonstrated clinically significant efficacy in pancreatic cancer. MSI-H is rare in pancreatic cancer (approximately 1% of patients). Some studies that have evaluated the efficacy of immunotherapy in different MSI-H tumours have shown modest activity in pancreatic cancer [8]. Despite this, the ability to screen for MSI-H tumours can identify a group of patients with pancreatic cancer in whom immunotherapy may be more effective.

To summarize, these are the key points: (i) the presence of mutations in DNA repair genes is a predictive biomarker of platinum-based chemotherapy efficacy; (ii) the presence of a germinal mutation in *BRCA1* or *BRCA2* is a predictive biomarker of olaparib efficacy; (iii) the presence of native *KRAS* allows the identification of a subgroup of patients with a higher probability of presenting a molecular alteration that may be a possible therapeutic target; and (iv) the presence of MSI-H is a predictive biomarker of response to immunotherapy.

### Biliary tract cancer

Chemotherapy based on cisplatin plus gemcitabine has been the standard first-line treatment for a decade in patients with BTC and has shown benefits over gemcitabine monotherapy, with a median OS of 11.7 months [15]. In recent years, new therapeutic options have been developed, both for first-line treatment and for more advanced lines of treatment. Among such options are new chemotherapy combinations, such as folinic acid, 5-fluorouracil and oxaliplatin (FOLFOX) [16]), capecitabine and irinotecan (XELIRI) [17] and gemcitabine/nab-paclitaxel plus cisplatin [18], as well as targeted therapies that are not guided by biomarkers, such as regorafenib [19].

**Fig. 1:**
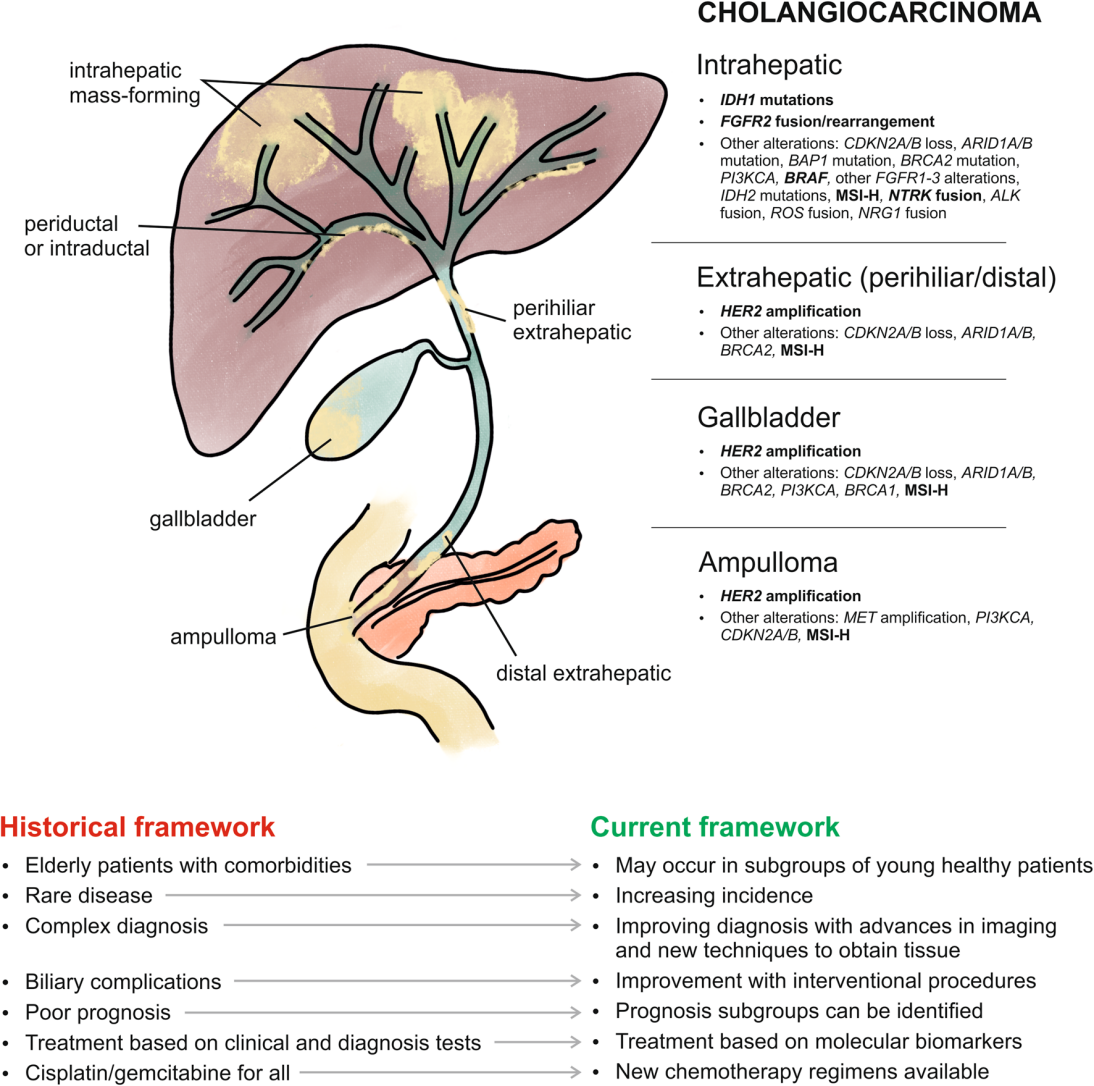
Historical and current framework of biliary tract carcinoma. *ALK* anaplastic lymphoma kinase, *ARID1A/B* AT-rich interactive domain 1A/B, *BAP1* ubiquitin carboxyl-terminal hydrolase BAP1, *BRAF* B-Raf proto-oncogene, *BRCA1* breast cancer gene 1, *BRCA2* breast cancer gene 2, *CDKN2A/B* cyclin dependent kinase inhibitor 2A/B, *FGFR1-3* fibroblast growth factor receptor 1-3, *FGFR2* fibroblast growth factor receptor-2, *HER2* human epidermal growth factor receptor 2, *IDH1* isocitrate dehydrogenase-1, *IDH2* isocitrate dehydrogenase-2, *MET* mesenchymal epithelial transition factor, *MSI-H* microsatellite instability-high, *NRG1* neuregulin 1, *NTRK* neurotrophic tyrosine receptor kinase, *PIK3CA* mesenchymal epithelial transition factor, *ROS* ROS proto-oncogene

BTC comprises a set of very heterogeneous tumours in multiple aspects, such as anatomical location, aetiology, clinical presentation, prognosis and surgical treatment. However, from the classical oncological point of view, all are grouped into a single entity. Fortunately, rapid advances in the molecular understanding of these tumours have revolutionized their screening and treatment (Fig. 1). BTC, especially the intrahepatic cholangiocarcinoma (IH-CCA) subtype, is a target-rich disease, from the molecular point of view, allowing for targeted therapies. Forty per cent of IH-CCA patients present targetable molecular alterations [20].

MOSCATO-01 was a “proof of concept” study that demonstrated the usefulness of conducting a molecular study in patients with BTC; 68% of the patients evaluated had treatable alterations, and 53% were candidates for matched targeted therapy, a percentage higher than that observed for the entire set of tumours. The study reported a disease control rate of 88% and a median OS of 17 months in patients treated with targeted therapy, compared to 5 months for patients who did not [21].

*NTRK* rearrangements and MSI-H, although infrequent in BTC (< 1%), are tumour-agnostic biomarkers for the use of tropomyosin receptor kinase inhibitors (i*TRKs*)*,* such as entrectinib and larotrectinib [20] and immune checkpoint inhibitors (ICIs) [22], respectively. Other alterations, such as *FGFR2* rearrangements or mutations (10-15%), B-Raf proto-oncogene (*BRAF*) *V600* mutations (3%), Human Epidermal growth factor Receptor 2 (*HER2*) amplifications or mutations (15%) and *IDH1* mutations (13%), are more prevalent, especially in patients with IH-CCA [22]. The combination of *BRAF* and mitogen-activated protein kinase (*MEK*) inhibitors (such as dabrafenib-trametinib) in patients with *BRAF V600E* [23] and, very recently, the double inhibition of *HER2* with trastuzumab and pertuzumab in patients with amplified *HER2* [24] have demonstrated efficacy in terms of the radiological response rate in nonrandomized phase I and II trials.

Of all the possible targets, *FGFR2* and *IDH1* are the most clinically important in BTC due to their incidence and pharmacological development. The ClarIDHy trial was the first positive phase III study that evaluated a biomarker-guided therapy in patients with BTC, resulting in FDA approval of ivosidenib in August 2021 for the treatment of patients with advanced or metastatic refractory CCA with *IDH1* mutations.

*FGFR2* inhibitors in patients with *FGFR2* rearrangements have also exhibited efficacy and safety in nonrandomized phase II clinical studies. Although there are multiple drugs in development, pemigatinib (approved by the FDA and the EMA) and infigratinib (approved by the FDA) are the most developed at the clinical level [25, 26].

In 2018, the European Society of Medical Oncology (ESMO) published a scale for the clinical performance of molecular targets (Scale for Clinical Actionability of Molecular Targets of the ESMO [ESCAT]), defining 6 levels of clinical evidence in relation to the therapeutic management of patients [27]. ESCAT level 1 indicates that the association between the alteration and the drug has been validated in clinical trials and therefore should guide therapeutic decisions. In 2020, based on the number of patients who would have to be analysed for each tumour to identify one that could benefit from ESCAT level 1 targeted treatment, ESMO’s Precision Medicine Working Group recommended routine next-generation sequencing (NGS) of four tumours types: cholangiocarcinoma (CCA), non-small cell lung cancer, prostate cancer and ovarian cancer [28].

## Anatomopathological aspects

### Anatomopathological diagnosis

To provide an adequate and effective treatment, especially for cases of unresectable neoplasias, it is necessary to make an anatomopathological diagnosis as accurately as possible through the identification of the origin of the primary neoplasia (pancreatic or biliary tract) or metastases.

#### Sample types

##### Pancreatic cancer

Samples can be obtained from biopsy, or by puncture of the primary pancreatic neoplasm, of the resection specimen or of the metastases.

In the vast majority of cases, the diagnosis is made by obtaining material from the pancreatic tumour by fine-needle aspiration (FNA) or by transgastric or transduodenal endoscopic ultrasound-guided fine-needle aspiration (EUS-FNA) [29]. Currently, biopsy is performed percutaneously or with core needle biopsy (CNB). As the material obtained is usually scarce, an attempt should be made to optimize collection as much as possible. Commercial fixatives should be used for sample preservation and fixation. Tissue fragments should be embedded in paraffin and sectioned for haematoxylin and eosin (H&E) staining, immunohistochemistry (IHC) and histochemistry. Remaining material should be processed for liquid cytology with Papanicolaou staining and for IHC, if necessary.

If the specimen extracted during a Whipple resection or cephalic pancreaticoduodenectomy is available, the histological diagnosis will already have been made, and the material obtained should be used for pathological staging and molecular diagnosis.

Finally, liver or lymph node biopsy obtained by transgastric or transduodenal EUS-guided FNA or CNB or percutaneous ultrasound-guided or computed tomography (CT)-guided FNA/CNB is usually used to confirm the diagnosis in patients with advanced neoplasia who are not candidates for initial surgery (by locoregional extension or by distance extension).

##### Extrahepatic biliary tract cancer

Samples can be obtained from primary neoplasms of the extrahepatic bile duct or from metastases.

If specimens are obtained by EUS-guided FNA/CNB, preparation similar to that described for pancreatic cancer specimens should be performed. If the sample is obtained by endoscopic retrograde cholangiopancreatography (ERCP), brush cytology should be used for processing; samples should also be prepared for Papanicolaou staining and IHC, if necessary. Finally, small biopsies can be obtained with cholangioscopy-guided microforceps biopsy by direct endoscopic imaging of intraductal lesions of the intrahepatic and extrahepatic bile ducts. These biopsy specimens should be fixed in 10% formaldehyde and processed as a biopsy.

##### Intrahepatic cholangiocarcinoma

Samples can be obtained from primary neoplasms of the intrahepatic bile duct or from metastases.

Depending on location, specimens can be obtained by percutaneous hepatic CNB or by EUS-FNA or EUS-CNB and prepared as tissue blocks or for cytology, with the material obtained by EUS processed similarly to that described for pancreatic cancer samples. If a specimen is obtained by ERCP, brush cytology should be used to process the material; the sample should also be processed for Papanicolaou staining and IHC, if necessary. Similarly, to that described for extrahepatic bile duct biopsies, small biopsies can be obtained with cholangioscopy-guided microforceps biopsy. These biopsy specimens should be fixed in 10% formaldehyde and processed as a biopsy.

#### Microscopic study

##### Pancreatic cancer

A histological diagnosis of pancreatic ductal adenocarcinoma (PDAC) is characterized by the formation of glandular structures lined by mucinous cubic cells surrounded by an abundant desmoplastic stroma. A histological grade (1, 2 or 3) is assigned based on glandular differentiation, mitotic activity, nuclear pleomorphism and mucin production [29, 30].

PDACs express cytokeratin 7 (CK7); however, CK7 is not an unequivocal IHC marker of PDAC because it is also expressed in the epithelium of the extrahepatic bile duct, gallbladder and neoplasms originating in these tissues. For this reason, a basic IHC panel that includes CK7, cytokeratin 20 (CK20), synaptophysin and trypsin should be performed for the differential diagnosis of other primary solid pancreatic neoplasms, especially well-differentiated neuroendocrine tumours (NETs), neuroendocrine carcinoma and acinar carcinoma, or of metastases of other neoplasms (Table 1).

**Table 1:** Immunohistochemical staining in the differential diagnosis of pancreatic cancer and histological subtypes of cholangiocarcinoma^a^

Pancreatic cancer	CK7	Synaptophysin	Trypsin
Ductal adenocarcinoma	+	−	−
Well-differentiated neuroendocrine tumour	−	+	−
Neuroendocrine carcinoma	+/−	+	−
Acinar cell carcinoma	+/−	+/−	+

Histological subtypes of cholangiocarcinoma	CK7	CK20	MUC2	MUC5	CDX2
Pancreaticobiliary	+	−	−	+	+
Intestinal	−	+	+	−	+

^a^Classification of digestive system tumours of the World Health Organization [64]

*CK7* cytokeratin 7, *CK20* cytokeratin 20, *MUC2* mucin 2, *MUC5* mucin 5, *CDX2* caudal-type homeobox 2

##### Extrahepatic biliary cancer

Most carcinomas are pancreaticobiliary adenocarcinomas, but there are other histological types, such as intestinal; these carcinomas should be differentiated by their prognostic and therapeutic implications. The IHC profile of different histological types of carcinomas allows them to be distinguished; for example, the pancreaticobiliary type expresses CK7 and mucin 5a (MUC5a), and the intestinal type expresses CK20, caudal-type homeobox 2 (CDX2) and mucin 2 (MUC2) (Table 1) [30].

##### Intrahepatic biliary cancer

Intrahepatic BTC is also called intrahepatic CCA, and there are two subtypes: large duct and small duct [33].

Large duct intrahepatic CCA is a perihilar tumour, with a morphology and IHC profile similar to that for extrahepatic CCA described in the previous section.

Small duct intrahepatic CCA is a peripherally located tumour that expresses immunohistochemical markers of cholangiolar differentiation, such as CK7, cytokeratin 19 (CK19) and epithelial membrane antigen. There are cases of patients who present with tumours combined with hepatocarcinoma (hepatocholangiocarcinoma) [33].

### Determination of biomarkers

#### Biomarkers of pancreatic cancer

The genetics of pancreatic cancer are characterized by a group of alterations in four genes in more than 90% of cases, with very high variability in genetic and epigenetic alterations as well as variable levels of genomic instability.

The main genes altered in pancreatic cancer are the *KRAS* oncogene and the tumour suppressor genes cyclin dependent kinase inhibitor 2A (*INK4A*), tumour protein 53 (*TP53*) and SMAD family member 4 (*SMAD4*). These four genes encode proteins involved in cell proliferation, and their alteration eliminates the control of quiescence, the state in which the vast majority of cells of the pancreas are found. *KRAS* alterations are considered to be nearly universal in pancreatic cancer because they are present in more than 90% of cases. These alterations can be identified in pancreatic intraepithelial neoplasia 1 (PanIN-1) and pancreatic intraepithelial neoplasia 2 (PanIN-2) lesions (premalignant); therefore, they constitute early alterations. These alterations can be detected in gastric juice, bile, faeces and circulating plasma, with limited sensitivity [31], and the determination of *KRAS* can be performed in tissue or cells using polymerase chain reaction (PCR).

The *INK4A* gene encodes p16 and p14 and is involved in the regulation of the cell cycle through the retinoblastoma pathway (p16) and in the regulation of genetic damage and cell cycle arrest through the p53 pathway (p14). This gene is inactivated in almost 100% of cases of pancreatic cancer and is detected in PanIN-2 lesions and carcinomas, indicating that inactivation occurs after rat sarcoma virus (*RAS*) alteration. Using IHC, p16 and p14 can serve as surrogate markers when differentiating among dysplasia, carcinoma or reactive changes. In these cases, IHC can be used to detect insulin-like growth factor II messenger ribonucleic acid (mRNA) binding protein 3 (IMP3), which in the pancreas and the bile duct is expressed in high-grade dysplastic lesions but not expressed in normal or reactive ducts and rarely in low-grade dysplasias [32].

Alterations in *TP53* are present in 50-70% of cases of pancreatic carcinoma and are only detected in PanIN-3 lesions and carcinomas. Alterations in the *SMAD4* gene are present in the protein encoded by the gene, which is involved in this pathway. Transforming growth factor beta (TGF-β) is inactivated in almost 60% of cases. Alterations in this gene are also exclusive to PanIN-3 and carcinomas; therefore, the loss of nuclear expression of *SMAD4*, as determined through IHC, helps to distinguish malignancies (*in situ* or invasive) from benign tumours. This alteration is very useful, especially in the identification of pancreatic cancer in biopsies of metastases of unknown origin [33].

In the small group of patients with wild-type or nonmutated *KRAS* pancreatic adenocarcinoma, mutations in other genes, such as *NTRK* and *NRG1*, are more likely to be found [34]. The frequencies of *NTRK* and *NRG1* fusions are 0.3 and 0.5%, respectively [35].

For both pancreatic cancer and biliary tract tumours, IHC, fluorescence *in situ* hybridization (FISH) and reverse transcription PCR (RT-PCR) can be used for screening [36]. Studies using pan-TRK monoclonal antibody mixtures have revealed positive *TRK* expression in tumour samples [37, 38], with a sensitivity of 75%. Up to 45% of tumours with *NTRK3* fusions can be negative by IHC [39]. False negatives may be related to sample preparation (e.g. during fixation). Similarly, positive results by IHC must be confirmed by a molecular method that verifies the presence of a fusion (e.g. FISH or NGS) to avoid the detection of overexpressed wild-type TRK proteins [40].

The study of repair protein genes (MutL homolog 1 [*MLH1*], MutS homolog 2 [*MSH2*], MutS homolog 6 [*MSH6*] and *PMS1* homolog 2, mismatch repair system component [*PMS2*]) has become routine in the daily analysis of biopsies. The loss of repair activity of these proteins results in a hypermutator phenotype. The sensitivity and specificity of two IHC panels for *PMS2* and *MSH6* is 100% for detecting mismatch repair (MMR) protein deficiency; therefore, a four-panel test is not strictly necessary [41, 42].

#### Biomarkers of biliary tract cancer

Defects in the MMR system are very rare in extrahepatic biliary tract carcinomas but are currently considered essential to identify Lynch syndrome. IHC should be employed to study repair proteins (*MLH1*, *MSH2*, *MSH6* and *PMS2*) and evaluate the possible loss of their expression that correlates with MSI in tumours.

Extrahepatic biliary tract carcinomas that overexpress *HER2/neu* are being described when using the same criteria as those used for breast cancer (3+ by IHC). Given its therapeutic implications, IHC for the detection of *HER2/neu* overexpression is recommended.

### Determination of biomarkers by next-generation sequencing

Tumour sequencing with high-efficiency techniques, such as NGS or “parallel NGS”, has been incorporated into oncological management [43]. They are fast and relatively low-cost techniques based on searching for molecular alterations in DNA and/or RNA fragments by PCR using a panel of genes chosen according to the type of tumour. These methods require little material, allow the detection of a high number of possible molecular alterations in a tumour with a single test and are applicable to blood and cytological samples of both fresh and paraffin-embedded tissue [44].

#### Method and steps

##### Study indication

Depending on the characteristics of each patient, procedures can be performed by either an oncologist or pathologist.

##### Sample selection

Clinicians should select an area for histological preparation with the following requirements: (i) a percentage of tumour cellularity greater than 10% with respect to the total cellularity of the tissue; (ii) no extensive areas of necrosis; and (iii) sufficient amount of the tumour in the sample to allow the extraction of at least 40 ng of DNA or RNA, for which ten 10-µm sections containing tumour can be made after adequate fixation and preservation. Depending on availability, in exceptional cases, for quantities as small as 10 ng, NGS can be performed.

##### Extraction of the material and preparation of libraries and sequencing

These procedures should be performed by a pathology technician supervised by a molecular biologist or biochemist. DNA is extracted and then amplified with DNA primers, allowing the simultaneous sequencing of multiple regions and the preparation of libraries for sequencing by PCR in second-generation sequencers.

##### Analysis and interpretation of results

Analysis and interpretation of the results require a molecular biologist or a biochemist in collaboration with a bioinformatician or software with remote support.

##### Integration of results

This should be performed by the pathologist with the multidisciplinary team responsible for the patient, in which an oncologist, a molecular biologist or biochemist, etc., serve as the core of a committee for tumours with molecular alterations (Molecular Tumour board) [44].

#### Recommended biomarker determinations based on current evidence

##### Pancreatic cancer

Table 2 describes the current clinical application of NGS in advanced ductal pancreatic carcinoma [28, 45]. Currently, there are several drugs in clinical development for advanced ductal pancreatic carcinoma (Table 3).

**Table 2:** Current clinical application of next-generation sequencing in the treatment of advanced ductal pancreatic carcinoma

Gen	Alteration	Prevalence	Method	Level of evidence ESCAT^1^/ASCO^2^	Drugs
*BRCA1*/2	Germinal mutation Somatic mutation	1–4% 3%	NGS NGS	IA^1^ Strong recommendation^2^ High quality of evidence^2^ IIIB^1^ Strong recommendation^2^ Low quality of evidence^2^	Olaparib Rucaparib
*NTRK*	Fusion	0.3–0.6%	NGS	IC^1^ Moderate recommendation^2^ Low quality of evidence^2^	Larotrectinib and entrectinib
*MLH1, MSH2,* *MSH6, PMS2* and *EPCAM*	MSI-H mutation	0.8–2%	NGS/IHC	Strong recommendation^2^ Low quality of evidence^2^	ICI (pembrolizumab)

*BRCA1/2* breast cancer gene 1 and 2, EPCAM epithelial cellular adhesion molecule, *ICI* immune checkpoint inhibitor, *IHC* immunohistochemistry, *MLH1* MutL homolog 1, *MSH2* MutS homolog 2, *MSH6* MutS homolog 6, *MSI-H* microsatellite instability-high, *NGS* next-generation sequencing, *NTRK* neurotrophic tyrosine receptor kinase, *PMS2* *PMS1* homolog 2, mismatch repair system component

^1^ESCAT scale for clinical actionability of molecular targets of the European Society for Medical Oncology (ESMO) [28]

^2^ASCO clinical practice guidelines [45]

**Table 3:** Drugs under development for the treatment of advanced pancreatic carcinoma

Gen	Alteration	Prevalence	Method	Level of evidence	Drugs
*KRAS*	Mutation (G12C)	90%	NGS/PCR	IIIA^1^	Adagrasib
*PIK3CA*	Access point mutation	3%	NGS	IIIA^1^	MK2206, alpelisib and buparlisib
*BRAF*	Mutation	3%	NGS	IIIA^1^	Dabrafenib and trametinib
*MDM2*	Amplification	2%	NGS	IIIA^1^	Nutlin-3
*ERBB2*	Amplification/mutation	1-2%	NGS	IIIA^1^	Trastuzumab and pertuzumab
*NRG1*	Fusion	1%	NGS	IIIA^1^	Afatinib
*ALK/RET/ROS1*	Fusion	< 1%	NGS	IIIA^1^	TPX-00005 and repotrectinib
*TMB*	TMB-H	10%	NGS	IIIA^1^	ICI (pembrolizumab)

*ALK* anaplastic lymphoma kinase, *BRAF* B-Raf proto-oncogene, *ERBB2* receptor tyrosine-protein kinase erbB-2, *ICI* immune checkpoint inhibitor, *KRAS* kirsten rat sarcoma virus, *MDM2* murine double minute 2, *NGS* next-generation sequencing, *NRG1* neuregulin 1, *PCR* polymerase chain reaction, *PIK3CA* phosphatidylinositol 4,5-bisphosphate 3-kinase catalytic subunit alpha, *RET* rearranged during transfection, *ROS1* ROS proto-oncogene 1, *TMB* tumour mutational burden, *TMB-H* tumour mutational burden-high

^1^ESCAT scale for clinical actionability of molecular targets of the European Society for Medical Oncology (ESMO) [28]

##### Biliary tract cancer

The percentage of “actionable” genetic alterations varies between intrahepatic, extrahepatic or gallbladder CCA [46]. Table 4 provides the current recommendations for the use of NGS in intrahepatic, extrahepatic and gallbladder BTC. Regarding BTC, the FDA has recently approved drugs that inhibit *IDH1* and *FGFR2*, genes that are most frequently altered in intrahepatic CCA, but which have also demonstrated efficacy in extrahepatic biliary tract tumours with the same genetic alteration. Other drugs for the treatment of intrahepatic, extrahepatic and gallbladder carcinoma of the bile duct are currently being studied (Table 5) [47].

**Table 4:** Current clinical application of next-generation sequencing in the treatment of intrahepatic, extrahepatic and gallbladder carcinoma of the bile duct

Gen	Alteration	Prevalence		Method	Level of evidence ESCAT^1^/ASCO^2^	Drugs
		EH and gallbladder	IH			
*IDH1*	Mutation	3%	10–20%	NGS	IA^1^ Strong recommendation^2^ High quality of evidence^2^	Ivosidenib
*FGFR2*	Fusion/rearrangement	1%	4–15%	NGS	IB^1^	Pemigatinib, infigratinib
*NTRK*	Fusion/rearrangement	2%	2%	NGS/IHC	IC^1^ Moderate recommendation^2^ Low quality of evidence^2^	Larotrectinib and entrectinib
*MLH1, MSH2,* *MSH6, PMS2, EPCAM*	Mutation (MSI-H)	0.5-2%	1%	NGS/IHC	IA^1^ Strong recommendation^2^	Pembrolizumab (aPD1)

*aPD1* anti-programmed cell death protein 1, *EH* extrahepatic, *EPCAM*: epithelial cellular adhesion molecule, *FGFR2* fibroblast growth factor receptor-2, *ICI* immune checkpoint inhibitor, *IDH1* isocitrate dehydrogenase-1, *IH* intrahepatic, *IHC* immunohistochemistry, *MLH1* MutL homolog 1, *MSH2* MutS homolog 2, *MSH6* MutS homolog 6, *MSI-H* microsatellite instability-high, *NGS* next-generation sequencing, *NTRK* neurotrophic tyrosine receptor kinase, *PMS2* *PMS1* homolog 2, mismatch repair system component

^1^ESCAT scale for clinical actionability of molecular targets of the European Society for Medical Oncology (ESMO) [28]

^2^ASCO clinical practice guidelines [45]

**Table 5:** Drugs under development for the treatment of intrahepatic, extrahepatic and gallbladder carcinoma of the biliary tract

Gen	Alteration	Prevalence		Method	Level of evidence ESCAT^1^/ASCO^2^	Drugs
		EH and gallbladder	IH			
IDH1	Mutation	3%	10–20%	NGS		HMPL-306, IDH-305, FT2012 (olutasidenib), AG881 (vorasidenib), LY3410738
*IDH2*	Mutation	1%	6%	NGS	IIIB^1^	Enasidenib, LY3410738, AG881 (vorasidenib)
FGFR2	Fusion/rearrangement	1%	4–15%	NGS	IB^1^	Futibatinib (TAS120), erdafitinib, derazantinib
*FGFR2*	Other alterations					Futibatinib (TAS120), erdafitinib, derazantinib
*NTRK*	Fusion/rearrangement	2%	2%	NGS/IHC	IC^1^ Moderate recommendation ^2^ Low quality of evidence^2^	LOXO-195 (selitrectinib) and TPX-00005 (repotrectinib)
MET	Amplification	2–3%	5%	NGS	IIIA^1^	Tivantinib/crizotinib
*PIK3CA*	Access point mutation	7%	6%	NGS	IIIA^1^	MK2206, alpelisib and buparlisib
*BRAF*	Mutation	3-5%	3%	NGS	IIB^1^	Dabrafenib and trametinib Belvarafenib
*BRCA1/2*	Mutations	3%	3–5%	NGS	IIIA^1^	Olaparib and rucaparib
*ERBB2*	Amplification/mutation	10–15%	7%	NGS/FISH	IIIA^1^	Trastuzumab and pertuzumab Zanidatamab Trastuzumab-deruxtecan
*TMB*	TMB-H	3%	6%-12%	NGS	IIIA^1^	Pembrolizumab

*BRAF* B-Raf proto-oncogene, *BRCA1/2* breast cancer gene 1 and 2, *EH* extrahepatic, *ERBB2* receptor tyrosine-protein kinase erbB-2, *FGFR2* fibroblast growth factor receptor-2, *FISH* fluorescence *in situ* hybridization, *ICI* immune checkpoint inhibitor, *IDH1* isocitrate dehydrogenase-1, *IDH2* isocitrate dehydrogenase-2, *IH* intrahepatic, *IHC* immunohistochemistry, *MET* mesenchymal epithelial transition factor, *NGS* next-generation sequencing, *NTRK* neurotrophic tyrosine receptor kinase, *PIK3CA* phosphatidylinositol 4,5-bisphosphate 3-kinase catalytic subunit alpha, *TMB* tumour mutational burden, *TMB-H* tumour mutational burden-high

^1^ESCAT scale for clinical actionability of molecular targets of the European Society for Medical Oncology (ESMO) [28]

^2^ ASCO clinical practice guidelines [45]

## Role of liquid biopsy

The difficulty of obtaining a tumour tissue sample has long been an obstacle in the management of patients with biliopancreatic tumours. Endoscopic ultrasound-guided pancreatic aspiration or ERCP cytology/aspirates of the bile duct provide low tumour cellularity, which at most confirms malignancy but frequently requires repetition of the procedure, with consequent morbidity and therapeutic delays. Even less effective is NGS-type molecular studies with such samples, yet these studies can be very relevant for therapeutic decisions. In a retrospective study that analysed 149 histological samples from patients with advanced CCA, the sample failure rate was 27%; that is, only 1 in every 4 samples was valid for NGS, mainly due to the lack of tumour content.

Although it is not yet considered a standard test, liquid biopsy (LB) has been in development for years with the aim of overcoming these obstacles. It consists of a rapid and noninvasive test based on the detection and analysis of tumour genetic material released (by shedding) into biological fluid (blood, urine, bile, etc.), which can be used to indicate the molecular heterogeneity of these tumours [48]. There are several types of LB in development, such as the analysis of circulating tumour cells (CTCs) or exosomes (extracellular vesicles of endosomal origin), and the study of circulating tumour DNA (ctDNA) is gaining strength. The potential clinical utilities of ctDNA are very broad. Among them are early diagnosis with a potential increase in detection of resectable and therefore curable stages, the detection of minimal residual disease (MRD) that allows the selection of patients who may benefit from adjuvant treatment, the early detection of recurrence, the real-time monitoring of the response or resistance to treatment and the ability to describe the intratumoural heterogeneity in tumours [49]. The use of ctDNA in advanced disease allows the identification of potential therapeutic targets, especially in BTC. The main limitation of this technique is the scarcity of ctDNA, which poses a risk to its sensitivity for biomarker detection.

Mody *et al.* studied ctDNA in 124 patients with advanced BTC (70% with IH-CCA) and found treatable alterations in 21% of patients (*BRAF* [2%]; *aERBB2* [5%]; *fFGFR2* [2%]; *mFGFR2* [2%]; and *mIDH1* [10%]) [50]. In general, the agreement described between LB and tissue is quite acceptable (60-100%) [51]. Triple comparisons have also been made between LB, primary tumour tissue and metastatic tissue [52], which revealed similar alterations; therefore, the authors concluded that any source would be valid if it were the only one available. Although the mutation detection rate seems optimal with LB (83% sensitivity for *IDH1*), the rate of *FGFR2* rearrangement detection decreases because DNA fragmentation can hinder the detection of these fusions. The use of more modern panels, with greater coverage, could make these results comparable. Thus, at the 2021 Symposium on Gastrointestinal Cancer of the American Society of Clinical Oncology (ASCO), the results of 174 NGS studies with tissue and LB from patients with advanced CCA were reported; interestingly, the percentage of actionable alterations found was higher in LB samples than tissue samples (33.1 *vs.* 23.2%, respectively) and for *fFGFR2* (11.3 *vs.* 3.4%, respectively) [53].

The use of ctDNA has allowed the description of the resistance mechanism of *fFGFR2* and m*IDH1* CCA treated with *FGFR* and *IDH* inhibitors [54, 55] and allowed the observation that in some patients, new resistance mutations can be overcome with new drugs in development.

In PDAC, in line with the recommendations regarding tissue samples, the guidelines of the National Comprehensive Cancer Network^®^ (NCCN) recommend using LB when a tissue sample is not available [56]. The recommendations are very similar to those for BTC. The agreement between ctDNA and tissue biopsy has been reported as 78% [57]. In a recent study, 357 LBs (ctDNA) from patients with advanced PDAC were analysed; treatable alterations, including *KRAS* (G12C), epidermal growth factor receptor (*EGFR*), ataxia telangiectasia mutated (*ATM*), myelocytomatosis oncogene (*MYC*), *BRCA*, Phosphatidylinositol 4,5-bisphosphate 3-kinase catalytic subunit alpha (*PIK3CA*) and *BRAF*, were found in 48% of patients [58]. Notably, 9% of LBs revealed mutations in homologous recombination genes, with potential therapeutic implications, such as the possibility of selecting a platinum-based first-line treatment [59] and the advantage of obtaining results in a matter of days.

Improvements in ctDNA collection techniques and their increasing accessibility in the health care field, together with new molecular-level therapeutic evidence, will probably expand the use of ctDNA by clinicians for biliopancreatic tumours, not only in advanced disease but also in earlier stages and with broader uses, such as MRD detection or response monitoring.

## Role of genetic counselling

It has been reported that 5-10% of PDACs are hereditary in origin [60]. In addition, retrospective studies show that approximately 55% of PDAC cases with a hereditary origin do not meet the clinical criteria for familial pancreatic cancer (presence of two or more first-degree relatives with PDAC) [61]. For this reason, since 2018, the NCCN and ASCO guidelines recommend a germinal study of predisposition to PDAC (*BRCA1*, *BRCA2*, *ATM*, *PALB2*, *MLH1*, *MSH2*, *MSH6*, *PMS2*, *CDKN2A*, *TP53* and *STK11*) for all patients with PDAC, regardless of stage, which can have consequent personal and family history implications, among which are PDAC screening programmes with magnetic resonance imaging (MRI) or annual EUS. In addition, since the publication of the POLO study [3], in which the benefit of maintenance treatment with olaparib compared to placebo was demonstrated in patients with metastatic PDAC and germline mutations in *BRCA*1/2 that had not progressed after a platinum-based first-line treatment, the determination of this biomarker before starting first-line treatment has been added as a recommendation in these guidelines.

Unlike other predictive somatic biomarkers of targeted therapy, the determination of germline *BRCA* mutations has implications for patients and their relatives that require appropriate counselling adapted to their personal circumstances, both before and after performing the test. This process has traditionally been carried out in Family Cancer Units, with staff (doctors, nurses and psychologists) adequately trained and accredited for this purpose. These consultations, classically nonurgent, have a waiting list that can extend from weeks to months.

After a diagnosis of metastatic PDAC, patients should receive counselling and undergo testing within days to plan first-line systemic treatment.

There is a changing trend when referring patients to the Family Cancer Unit for accidental findings of pathogenic variants with high allelic frequencies (approximately 50%) obtained in somatic studies over the classic criteria of family history. However, currently, screening by this method alone is not considered adequate. In a study in which 187 patients with PDAC (not selected for their oncological family history) were examined both at the germinal and somatic levels, germline mutations were not detected in 8% of the patients in the somatic test [62].

This group of experts recommends that each medical oncology service develop a process, on the basis of its capabilities, that allows the adequate assessment, at the germinal level, of the largest possible number of patients with a recent diagnosis of advanced PDAC and obtain results within a period of days or, at most, weeks.

## Conclusion

Pancreatic cancer and BTC represent two tumours of low incidence but high mortality, with very poor oncological treatment outcomes. The most important advances have come from personalized medicine. For this reason, it is important to agree on the biomarkers recommended for use for these neoplasms.

In pancreatic cancer, this group of experts from the SEOM and the SEAP recommends determining, as a predictive marker, germinal mutations in the *BRCA* 1 and 2 genes (level of evidence IA) because they are associated with a greater response to treatment with platinum and PARP inhibitors [3, 4]. MSI-H, present in only 1% of patients with pancreatic cancer, should be assessed, with a strong recommendation, because as in other tumours, it is a predictive biomarker of response to immunotherapy. Furthermore, this group recommends determining the presence of *NTRK* and *NRG1* fusions*,* which are present in 0.3% and 0.5% of patients, respectively, with level of evidence IC [35], because the FDA has approved larotrectinib, a selective *TRK* inhibitor [63]. This group of experts considers it advisable to determine the presence of *KRAS* mutations, which are present in 93-95% of patients with pancreatic cancer. *KRAS* is not in itself a predictive biomarker, but tumours with native *KRAS* can identify a group of patients with the greatest possibility of presenting a molecular alteration that may be a possible therapeutic target.

BTC comprises a set of very heterogeneous tumours in multiple clinical and molecular aspects. Forty per cent of IH-CCA patients present targeted molecular alterations [20]. In the bile duct, there is a clear recommendation for the determination of the presence of *IDH1* gene mutations (level of evidence IA), which are present in 3% of cases, and the fusion or rearrangement of *FGFR2* (level of evidence IB), which are present in 4-15% of patients. These alterations have specific targeted treatments, such as ivosidenib and pemigatinib [6]. As in pancreatic cancer, it is recommended to determine the presence of *NTRK* rearrangements (recommendation IC) and MSI-H (recommendation IA); although their presence is infrequent in BTC (<1%), they are tumour-agnostic biomarkers that indicate the use of *NTRK* inhibitors*,* such as entrectinib and larotrectinib [20], and ICI inhibitors [22], respectively.

Tumour sequencing with high-efficiency techniques such as NGS allows detection of a high number of possible alterations with a single test that requires little material [44]. This consensus, similar to the consensus of the ESMO Precision Medicine Working Group, recommends the routine performance of NGS for CCA. Given the difficulty of obtaining a tumour tissue sample from these tumours, LB is an alternative because it has high agreement with tissue biopsy, offering multiple potential clinical uses. The development of personalized medicine in these tumours will allow deepening the knowledge about and application of new targeted therapies.

## Data Availability

Not applicable.
